# 561. Microbial Analysis and Fungi-Bacteria Correlation in Chronic Wound Infection Patients

**DOI:** 10.1093/ofid/ofad500.630

**Published:** 2023-11-27

**Authors:** Qingqing Wang, Bijie Hu

**Affiliations:** Zhongshan hospital, Fudan University, Shanghai, Shanghai, China; Department of Infectious Diseases, Zhongshan Hospital, Fudan University, Shanghai, Shanghai, China

## Abstract

**Background:**

Chronic wound infection has become a major healthcare burden globally. The colonized microbiome features and correlation between bacteria and fungi have not been listed.

**Methods:**

In this study, we enrolled 38 acute wound infection and 28 chronic wound infection patients. The wound swabs sampling at admission were tested by metagenomic next-generation sequencing. Microbial community characteristics in wound infection patients and bacteria-fungi correlations in chronic wound infection patients were analyse after filtering background organisms.

**Figure 1.**
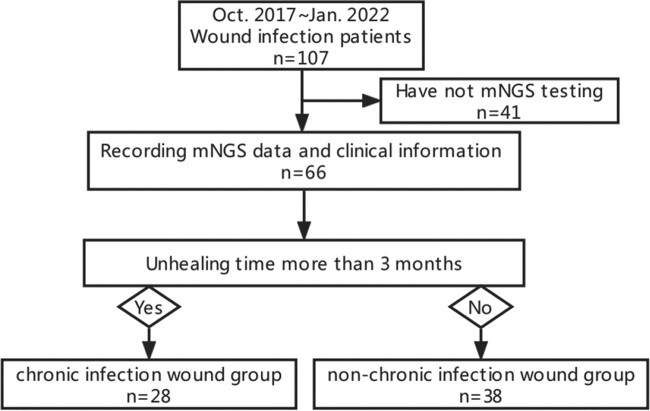
Flow chart

**Results:**

We found that wound microbiome in chronic wound infection patients had higher abundance of Pseudomonas aeruginosa than that in acute wound patients. Meanwhile, microbes with high relative abundant in chronic infection wounds were less significantly association with plasma inflammation factors compared with those in acute infection wounds. Finally, we investigated the association between fungi and bacteria in chronic infection patients and found that relative abundance of Pseudomonas aeruginosa was negatively associated with that of Candida albicans.

**Figure 2.**
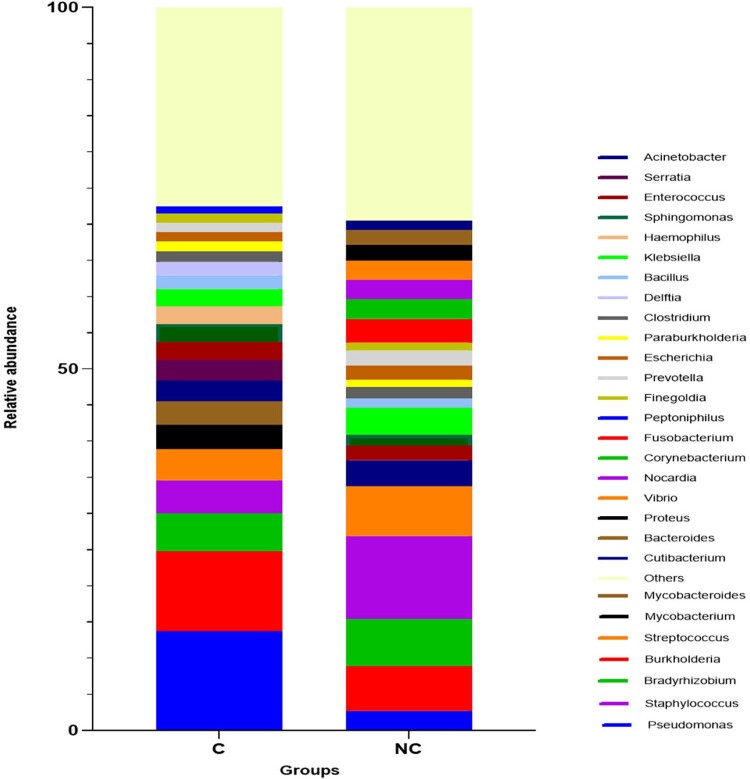
Comparison of bacteria species relative abundance between groups. Barplot of average relative frequencies of genera. Only genera>1% of relative abundance in wound were represented. C= chronic infection wound group, NC= acute infection wound group

**Figure 3.**
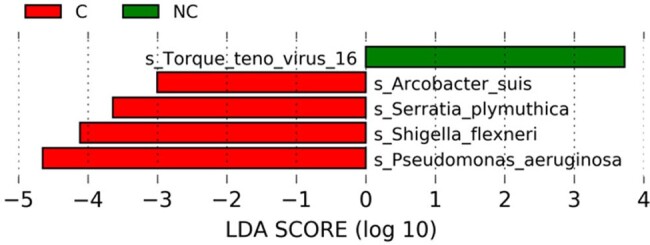
Analysis of Linear Discriminant Analysis (LDA) Effect Size in groups. The bar graph showed pathogens with LDA score log10 >5 in two groups. C= chronic infection wound group, NC= acute infection wound group.

**Figure 4.**
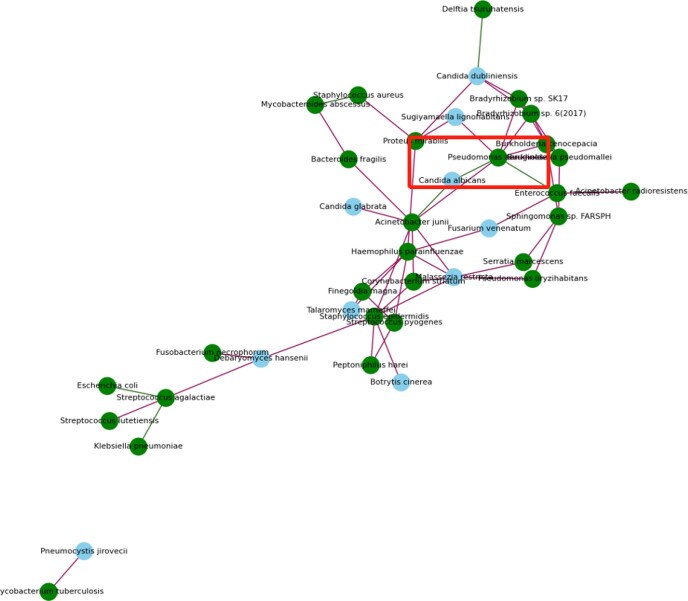
Fungi-bacteria network in patients with chronic infection wound. Green dots: bacteria; Blue dots: fungi; red edge: positive correlation; green edge: negative correlation. Relative abundance of Pseudomonas aeruginosa was negatively associated with that of Candida albicans highlighted with red box.

**Conclusion:**

Our metagenomic sequencing results of wound supported that Pseudomonas aeruginosa may be the biomarker of chronic wound infection and have interaction with candida albicans.

**Disclosures:**

**All Authors**: No reported disclosures

